# Supplementary material for: Changes on Techno-Functional, Thermal, Rheological, and Microstructural Properties of Tef Flours Induced by Microwave Radiation—Development of New Improved Gluten-Free Ingredients
Source: Foods. 2023 Mar 22;12(6):1345. doi: 10.3390/foods12061345 (PMC10048664; doi:10.3390/foods12061345)
Supplement: Supplementary file 1 [file foods-12-01345-s001.zip › foods-2282901-supplementary.pdf]

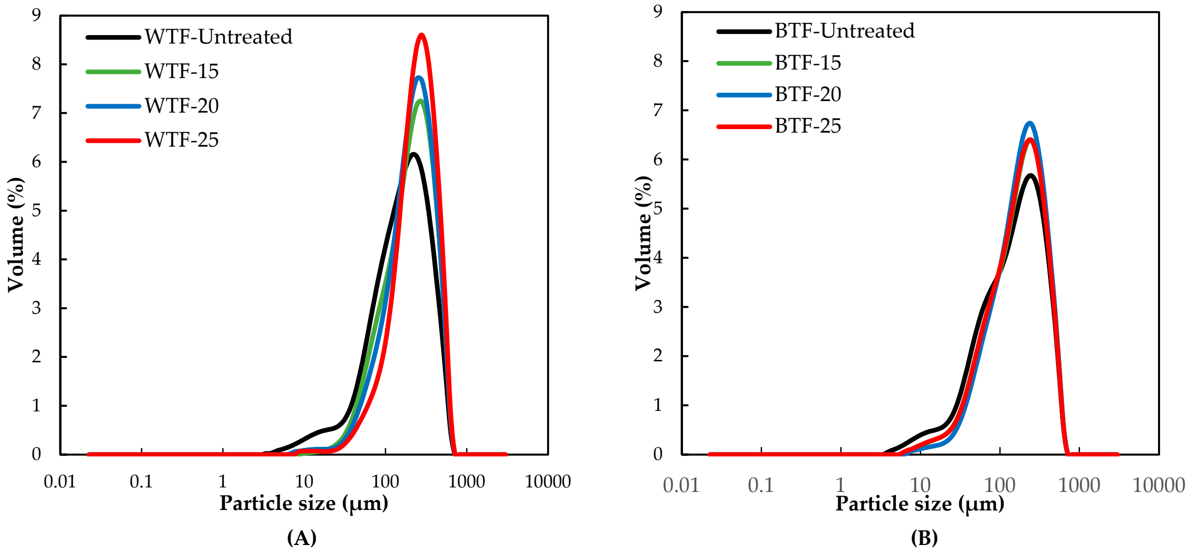

Figure S1. Particle size distribution of untreated and MW-treated tef flours at different moisture content (15%, 20%, and 25%). (**A**) White tef flour (WTF) and (**B**) brown tef flour (BTF).

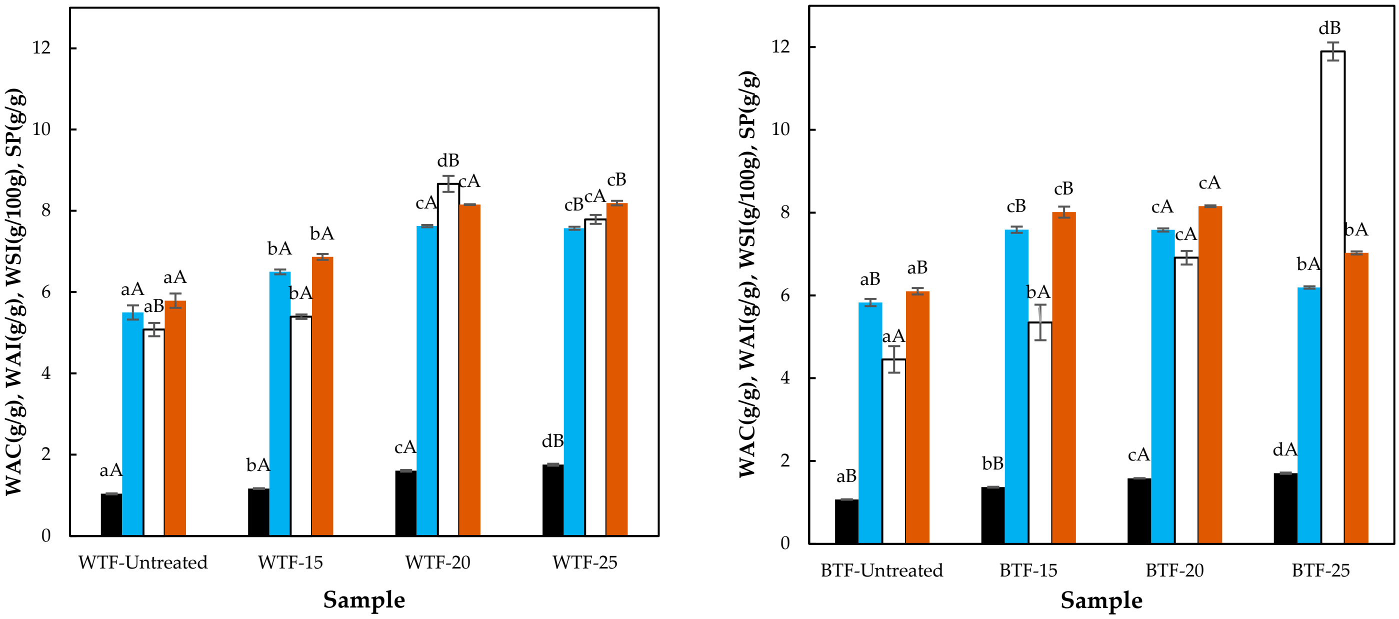

(A) **(B)**

Figure S2. Hydration properties of untreated and MW-treated tef flours at different moisture content (15%, 20%, and 25%). (**A**) White tef flour (WTF) and (**B**) brown tef flour (BTF). WAC (water absorption capacity, g water/g flour dry matter) (black), WAI (water absorption index, g sediment/g flour dry matter) (blue), WSI (water solubility index, g soluble solids/100g flour dry matter) (white), and SP (swelling power, g sediment/g insoluble solids flour dry matter) (brown). Different letters in the corresponding column within each studied factor indicate statistically significant differences between means at $p<0.05$. Lowercase letters are used to compare the effect of moisture content and capital letters to compare the effect of tef ecotype.

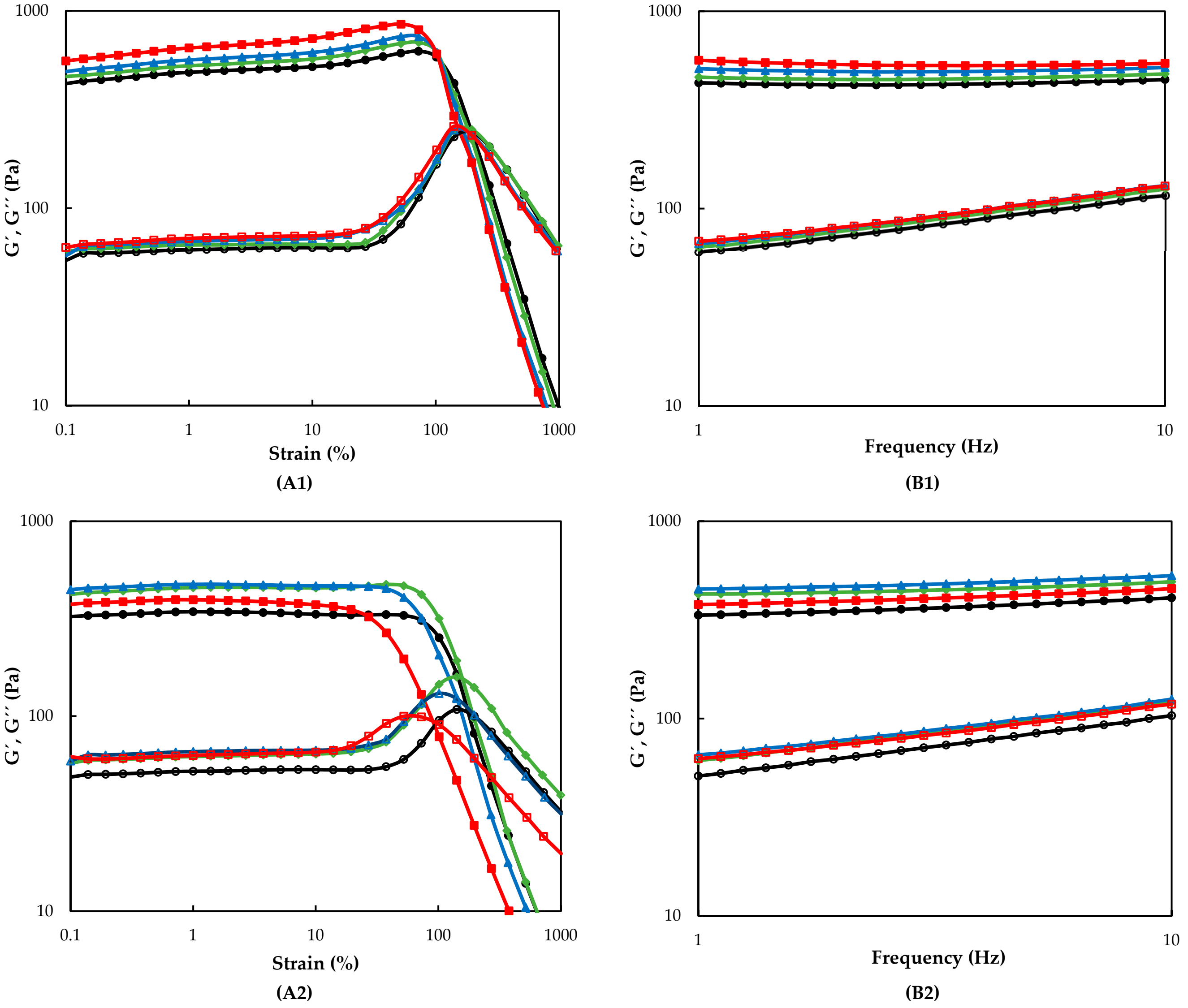

Figure S3. Strain sweeps (**A**) and frequency sweeps (**B**) of gels prepared from untreated white (1) and brown (2) tef flours (●○), and 15% (◆◇), 20% (▲△), and 25% (■□) MW-treated samples. G′ is represented by full symbols; G″ is represented by empty symbols.
